# Papaya Leaf Extract Elevates Platelet Levels in Individuals With Dengue Fever

**DOI:** 10.7759/cureus.61090

**Published:** 2024-05-26

**Authors:** Raymond Haward, Sonal Konjeti, Joshua Chacko, Jaya Sai Nadella, Simhadri Lakshmi Roja, Jaideep J Rayapudi

**Affiliations:** 1 Internal Medicine, Vydehi Institute of Medical Sciences and Research Centre, Bangalore, IND; 2 Medicine, Jawaharlal Nehru Medical College, Bangalore, IND; 3 Internal Medicine, Father Muller Medical College, Bangalore, IND; 4 Internal Medicine, Konaseema Institute of Medical Sciences and Research Foundation, Bangalore, IND; 5 Internal Medicine, NRI Medical College and General Hospital, Bangalore, IND; 6 Artificial Intelligence, Foresight Health Solutions, Chennai, IND; 7 Physiology, Pondicherry Institute of Medical Sciences, Pondicherry, IND

**Keywords:** acute dengue, non-severe dengue, natural medicine, high fever, carica papaya seeds, carica papaya (c. papaya) extract, carica papaya leaf extract, dengue thrombocytopenia, dengue fever/complications, thrombocytopenia

## Abstract

Dengue, an arboviral illness, is notorious for inducing thrombocytopenia, leading to bleeding and heightened mortality risk.* Carica papaya* leaf extract has shown efficacy in elevating platelet counts. A 35-year-old male presented with fever, fatigue, and body pain persisting for four days. Additionally, he complained of severe back pain, ocular discomfort, and brief episodes of nosebleeds. Testing revealed a positive NS1 antigen, prompting the initiation of intravenous normal saline, paracetamol, and papaya extract tablets. Despite initial platelet levels of 74,000, a subsequent decline to 30,650 was observed following another nosebleed. Subsequently, the patient's spouse administered freshly prepared papaya leaf extract orally three to four times daily, resulting in a platelet count of 120,320 on day 14. Timely recognition of declining platelet levels and the commencement of *C. Papaya* leaf extract contributed significantly to averting mortality risks.

## Introduction

The dengue virus (DENV), which causes dengue, belongs to the Flaviviridae family and has four distinct serotypes: DEN1, DEN2, DEN3, and DEN4 [1]. Female Aedes mosquitoes transmit these serotypes, which are characterized by severe muscle and retro-orbital pain [2]. The incidence of this disease rises during India's rainy season, from July to September, due to increased breeding sites for Aedes mosquitoes in the humid environment [2]. Dengue manifests with symptoms like headache, high fever, muscle and joint pains, and rashes, with a case fatality rate of less than 1%, while infection with one serotype confers lifelong immunity to it but only temporary immunity to others [1,2]. Later infections with different serotypes cause dengue hemorrhagic fever, a perilous condition characterized by low blood pressure, vascular leakage, and reduced platelet counts, with mortality rates ranging from 12% to 44% [2].

While viral fevers typically treat patients symptomatically in India and many other countries, dengue patients frequently receive papaya leaf juice to boost their platelet counts [3]. Recognizing its potential, the pharmaceutical industry has begun manufacturing papaya leaf extract medications for convenience. The process involves thoroughly washing fresh papaya leaves, cutting them into smaller pieces, blending them with a little water, and then straining the mixture to obtain smooth juice [4]. Some patients opt to enhance the juice's taste by adding honey or sugar [4]. This case highlights the significance of papaya juice in raising platelet counts.

## Case presentation

On a rainy day in August, a 35-year-old patient with no previous comorbidities presented to the office with complaints of fever, fatigue, and body pain for the past four days. He has severe back pain, as well as pain behind the eyes. The fever was continuous and associated with severe generalized body pain. The patient experienced bleeding from the nose for a minute the previous day. The patient denied any prior hospitalization and had been taking paracetamol for the past four days. He also complains of a reduced appetite. Family history revealed that the father died of a heart attack at age 70 and the mother died of breast cancer metastasis at age 68. The patient has no allergic history to any medications, has smoked one pack for the past 10 years, and occasionally drinks alcohol on weekends. 

Examination

The patient was a middle-aged male, ill-looking, well-nourished, and oriented with time, place, and person. He had a blood pressure of 108/78 mmHg, a pulse of 85/min, oxygen saturation of 96%, and a respiratory rate of 22/min at the time of examination. He was febrile (101°F). The liver was firm, non-nodular, and enlarged 2 cm below the right costal margin, while his abdomen was soft, non-tender, and mildly distended. During chest auscultation, there were decreased breath sounds above the right base. The examination of the cardiovascular system was unremarkable. We admitted the patient, sent her blood work, and discovered that her platelets were 74104/mm^3^, and his NS1 test came back positive (Table 1).

**Table 1:** Laboratory Investigation Results MCH: mean corpuscular hemoglobin; MCHC: mean corpuscular hemoglobin concentration; MCV: mean corpuscular volume HCT: hematocrit; ALP: alkaline phosphatase; GGT: gamma-glutamyl transferase; AST: aspartate aminotransferase; ALT: alanine transaminase

Laboratory investigation	Reference value	1st day	3rd day	5th day	7th day	14th day
Erythrocytes (*10^12^/L)	4-5.9	4.21	4.32	4.3	4.28	4.6
Hemoglobin (g/L)	12-16	13.6	13.4	13.5	13.4	13.5
MCV (fL)	80-100	96.2	94.3	95.1	93.6	94.7
MCH (pg)	27-31	29.1	29.4	28.7	29.3	29.2
MCHC (g/L)	32-36	31.2	31.1	32.7	33.8	32.8
HCT (%)	40-54	34.1	34.7	34.3	33.1	33.8
Leukocyte (*10^9^/L)	4.5-11	3.74	3.01	2.91	2.71	4.4
Neutrophils (%)	50-70	47	42	43	44	58
Eosinophils (%)	0.5-5	0.2	0.5	1	1.2	1.4
Basophils (%)	0-1	0	0.7	0.5	0.6	0.7
Lymphocyte (%)	20-45	46.8	48.6	49.3	52.5	35
Monocyte (%)	3-8	6	5.4	4.8	6.7	4.9
Thrombocyte (*10^9^/L)	150-400	74.1	30.65	27.45	38.65	120.3
Serum creatinine (mg/dL)	0.6-1.2	0.7	0.8	0.6	0.8	0.77
Albumin (g/dL)	3.5-5.5	3.5	4	4.2	4.1	3.9
Total bilirubin (mg/dL)	0.3-1	1.4	1.2	1	0.9	1.1
Unconjugated	0.2-0.8	1.1	0.9	0.6	0.6	0.4
Conjugated	0.0-0.3	0.3	0.3	0.4	0.3	0.7
ALP (IU/L)	40-129	113.5	110.6	107.4	104.7	112.6
GGT (IU/L)	8-61	24	32	28	30	26
AST (U/L)	8-48	49.3	47.9	44.6	39.6	39.2
ALT (U/L)	7-55	53.7	51.2	40.6	42.2	40.2
Glucose (mg/dL)	70-110	100	105	98	94	94
Total protein (g/dL)	6.2-8	6.8	6.75	6.5	6.3	6.25

Initially, the patient received fluid therapy with 0.9% normal saline, administered at a rate of 20 mL/kg/hr for the first two hours, followed by 10 mL/kg/hr for the subsequent six hours. Additionally, oral paracetamol was continued at a dosage of 650 mg, and Caripill was initiated at 1100 mg three times daily. The medical team also advised the patient to consume a lot of water. Over the next two days, the patient's platelets declined to 30650/mm^3^, and she had one more episode of bleeding from her nose for five minutes, necessitating a platelet transfusion. At this time, the patient's wife was insistent on trying the papaya leaf juice, which she made by collecting leaves from her home, and the doctor agreed. His wife used three papaya leaves per session, cutting them into smaller pieces with scissors. She then added 200 mL of water to a blender, blended the mixture, and strained the juice. The spouse administered this juice to the patient three to four times a day. On day 5, the platelets plummeted to 27450/mm^3^, but the rate of fall has drastically decreased. On day 7, the platelets increased to 38,654/mm^3^, and the patient's body pain started to decrease. The platelets increased continuously until they reached 120,320/mm^3^ on day 14, and the patient's symptoms resolved completely, with the only complaint being mild fatigue. After discharge and follow-up the following week, the patient's platelets reached 174,536/mm^3^. The patient thanked the team for their care.

## Discussion

Papaya leaf juice is known to increase platelet levels. Patients consuming papaya leaf extract exhibit a 15-fold increase in ALOX-12 genes, leading to an increase in megakaryocytes [5]. A study from Temple University reveals that ALOX12 targets a RUNX1 transcription factor in megakaryocytes and platelets. This raises the expression of genes that are unique to hematopoiesis [6]. Additionally, the papaya leaf extract, known to increase the platelet count, also increases the expression of the PTAFR gene [7]. However, when PTAFR induced platelets only to a certain level in mice, further administration did not increase the platelet count [7]. It may be due to auto-sensitization. Nevertheless, the PTAFR releases platelets and causes thrombocytosis [7].

Yunita et al.'s randomized control trial showed that this extract's capsules significantly raised the platelet count [8]. Besides an increase in platelet count, papaya leaf extract increases white blood cells [9]. Studies on patients with chemotherapy-induced thrombocytopenia also showed promising results in successful visits after taking *Carica papaya* leaf extract as a therapy for the ailment [9]. Studies on murine models revealed hematopoiesis and thrombopoiesis in the leaf extract, with a statistically significant increase in platelet levels and RBC count [10].

Also, papaya leaf extract makes pancreatic beta cells release more insulin, raises the pH of the stomach (which is good for digestion), stops bacteria from making proteins, and fights cancer by raising p53, Bax, PARP, and cleaved caspase-3 and lowering Bcl2 and MMP2/9 expression [11]. Having higher levels of ALOX-12 and PTAFR expression and lower levels of CCL6/MRP-1, CCL8/MRP-2, CCL12/MCP-5, and IL1R1 expression helps fight dengue [11]. The leaf extract also lowers proinflammatory cytokines like IL-1α, IL-1β, IL-6a, and IL-8. It also boosts anti-tumor immunity by raising IL-12p40, IL-12p70, IFN-γ, and TNF-α [11].

DENV replication in the host necessitates the inhibition of interferon signaling. Researchers have discovered that DENV can replicate efficiently in AG129 mice, which lack α-, β-, and γ-interferon receptors [12]. Using these laboratory models, we need to further explore DENV replication in conjunction with papaya leaf extract to determine any potential correlations. Notably, endothelial injury in liver tissues has been significantly associated with DENV, suggesting that this may be the site of viral replication [12].

Recent studies on various papaya formulations have raised safety concerns. Papaya is known to cause nausea and gastrointestinal upset and can interact with hypoglycemic agents, anti-malarial drugs, cardiovascular drugs, and antibiotics [12]. Additionally, the solvents used in the production of leaf extract capsules by various companies may affect the efficacy and side effects of these medications. We also need to carry out additional research on the pesticides and fertilizers sprayed on papaya leaves or applied to papaya trees.

Further research on flavonoids in leaf extracts is necessary. Flavonoids exhibit antiviral effects against herpes simplex, polio, parainfluenza, and respiratory syncytial viruses. For instance, flavonoids derived from fingerroot trees have shown competitive inhibition of the NS3 protease [12]. Researchers recognize the membrane-stabilizing effects and anti-sickling properties of flavonoids in *C. papaya* leaf extract [13]. These properties might help maintain and improve platelet counts. Continued research on *C. papaya* leaf extract will enhance our understanding of its impact on platelets and could lead to treatments for various ailments.

## Conclusions

This case and various studies demonstrate that papaya leaf extract effectively increases thrombocytes. When used in dengue, a viral disease that is usually treated by treating the symptoms, this* C. papaya* leaf extract has shown promise in raising platelet counts and lowering death rates in dengue patients.
